# Central retinal artery occlusion following transcatheter aortic valve implantation (TAVI) for aortic stenosis: a case report

**DOI:** 10.1186/s12886-026-04805-w

**Published:** 2026-04-29

**Authors:** Pablo Ballester Dolz, Kemal Mutibayraktaroglu, Yuri Belitsky, Philipp Schwember, Madeleine Zetterberg

**Affiliations:** 1 Department of Ophthalmology, Sahlgrenska University Hospital, Mölndal, Region Västra Götaland, Sweden; 2 Department of Clinical Neuroscience and Ophthalmology, Institute of Neuroscience and Physiology, Sahlgrenska Academy, University of Gothenburg, Gothenburg, Sweden

**Keywords:** Transcatheter aortic valve implantation, Central retinal artery occlusion

## Abstract

**Background:**

Transcatheter aortic valve implantation is the standard treatment for high-risk elderly patients suffering from symptomatic severe aortic stenosis. While this procedure is significantly less invasive compared to traditional methods, there are concerns regarding the potential risk of cerebrovascular accidents and ocular complications. We report a case of monocular central retinal artery occlusion that occurred following transcatheter aortic valve implantation due to aortic stenosis. The occurrence of central retinal artery occlusion post-transcatheter aortic valve implantation has not previously been reported.

**Case presentation:**

A 78-year-old male presented with a sudden, painless loss of vision in the left eye immediately after undergoing transcatheter aortic valve implantation. Fundus examination revealed moderate optic disc edema, mild venous tortuosity and diffuse retinal pallor at the posterior pole, most marked in the perimacular region and extending along the major vascular arcades into the mid-peripheral retina, with a cherry-red spot at the fovea. Optical coherence tomography indicated signs of retinal ischemia. The patient was referred to the emergency department for further assessment.

**Conclusions:**

Although there is evidence of embolization occurring after TAVI, this is the first case of central retinal artery occlusion that has been reported. Our findings underscore the need for thorough pre-TAVI counseling, increased neurologist awareness of this complication, and prompt ophthalmologic evaluation in cases of sudden monocular visual loss.

## Background

Aortic stenosis (AS) is the most common valvular disease in developed countries, affecting around nine million people globally [1]. Transcatheter aortic valve implantation (TAVI) is an established therapeutic approach for patients with severe symptomatic aortic stenosis, applicable to both high-risk and intermediate-risk groups [2, 3]. 

Previous research indicates that the risk of retinal embolization during or following interventional procedures, such as coronary bypass grafting, cardiac catheterisation (including TAVI), or carotid artery stenting, ranges from 55% to 100% after coronary bypass surgery, approximately 1.25% to 13.2% after carotid artery stenting, and around 6.3% after cardiac catheterisation [4]. 

Although TAVI is considerably less invasive than conventional surgical valve replacement, recent studies propose that it has a relatively high rate of cerebrovascular accidents, reaching up to 5% [2, 5]. 

Therefore, it has been suggested that a risk for retinal arterial occlusive events exists. Indeed, there are studies showing that retinal embolic events and the appearance of new retinal abnormalities following TAVI occur in 15% and 20% respectively [4], and that there is a noted impairment of choroidal microperfusion in patients undergoing TAVI [6]. We here present a case of central retinal artery occlusion (CRAO) following TAVI in a patient with aortic stenosis.

A literature review was conducted using PubMed and Google Scholar with the search terms: TAVI, CRAO, BRAO, retinal embolic events, and retinal ischemia and no previous reports were found.

## Case presentation

A 78-year-old man with a medical history of chronic ischemic heart disease (artherosclerosis, angina pectoris and PCI [percutaneous coronary intervention] in 2013) and aortic stenosis arrived at the ophthalmological emergency department at Sahlgrenska University Hospital. The visit was prompted by a referral from the cardiology department due to a sudden and significant reduction in vision that occurred immediately following TAVI, six days prior to presentation. A Naviator Transcatheter Aortic Valve 27 mm was implanted by transfemoral approach and balloon aortic valvuloplasty (BAV) was performed. A single dose of Heparin 8000IU was administered, and daily 75 mg acetylsalicylic acid was prescribed. A provisional pacemaker was used for telemetry throughout the post-operative period. Immediately after the TAVI procedure, the patient experienced a sudden decrease in vision in the left eye, followed by formed visual hallucinations which was reported to the cardiologist in the cardiac care unit. No other neurological symptoms were present. A brain CT scan was performed one day after TAVI and did not demonstrate an acute ischemic stroke but revealed older lacunar infarcts, including one adjacent to the head of the caudate nucleus, an older right frontal subcortical ischemic lesion, and moderate white matter changes.

The patient was hospitalized for three days with telemetry monitoring and was subsequently discharged home with an outpatient follow-up scheduled with an ophthalmologist.

He was known to the ophthalmology clinic due to blindness in the right eye secondary to a chronic retinal detachment that was initially diagnosed 11 years previously, resulting in secondary glaucoma. No retinal surgery was performed at the time of the diagnosis. In addition, the subject underwent cataract surgery in both eyes, approximately 16 years before presentation, and he also had Nd: YAG capsulotomy in the left eye three years after cataract surgery.

At presentation, visual acuity was no light perception (NLP) in the right eye and hand movement (HM) in the left eye (BCVA). Intraocular pressure measured with iCare tonometry was 41 mmHg in the right eye and 8 mmHg in the left eye. The patient denied ocular pain, and extraocular movements were normal.

The direct pupillary light reflex in the left eye was present; however, the indirect response and assessment of a relative afferent pupillary defect (RAPD) could not be performed. Slit-lamp examination of the anterior segment revealed an opaque cornea preventing further view of the right eye and no signs of inflammation in the left eye.

**Fig. 1:**
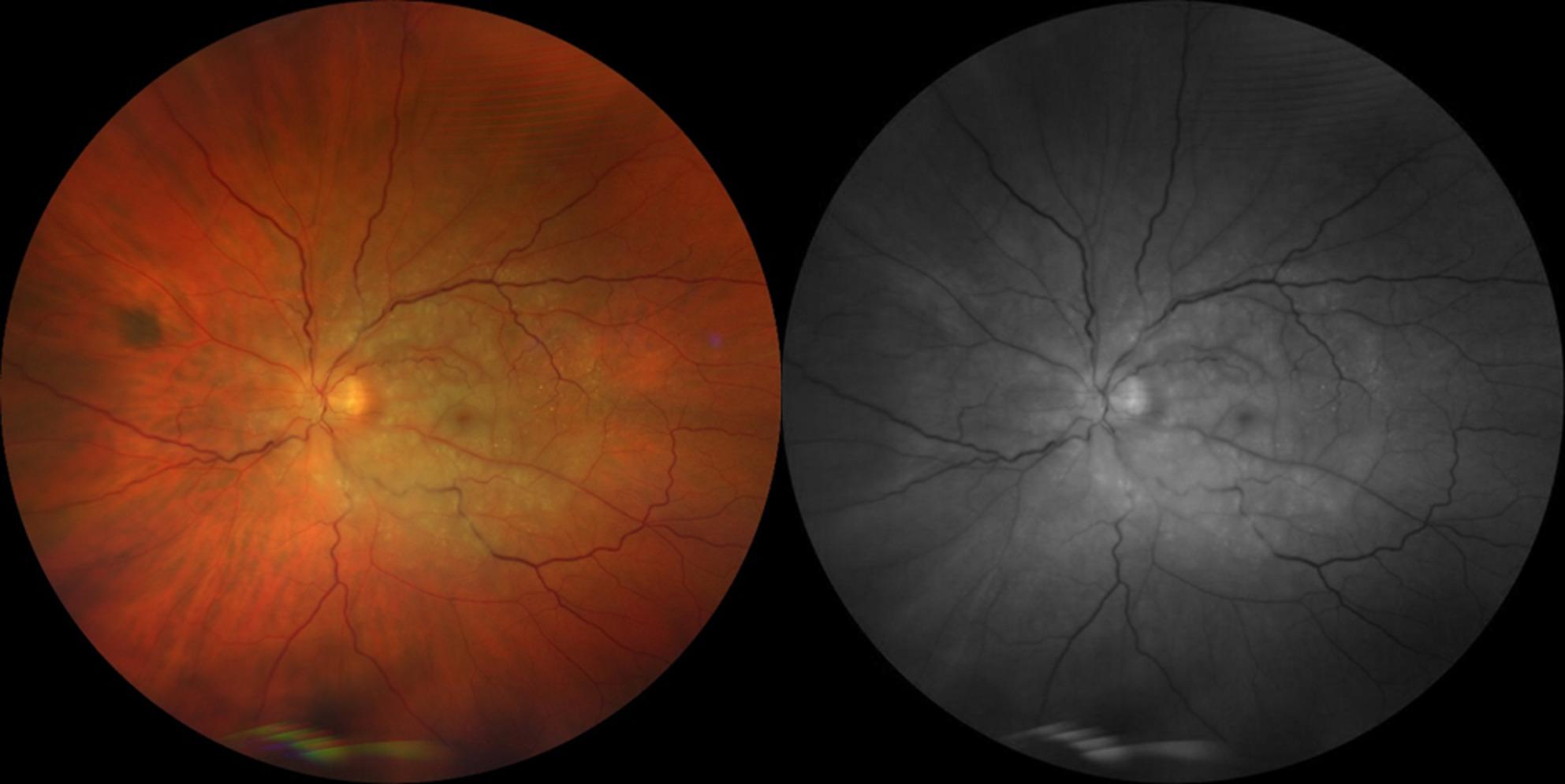
Color and green filter fundus pictures illustrate moderate optic disc edema with mild venous tortuosity and a diffusely pale posterior pole particularly in the perimacular region, with pallor extending along the major vascular arcades into the mid-peripheral retina and a cherry spot in the foveal area

Dilated examination showed a well-positioned intraocular lens with no evidence of vitritis or hemorrhage in the vitreous cavity. Fundus examination demonstrated moderate optic disc edema with mild venous tortuosity and a diffusely pale retina at the posterior pole, particularly in the perimacular region, with pallor extending along the major vascular arcades into the mid-peripheral retina and a cherry spot in the foveal area. Fundus photographs were obtained using a Zeiss Clarus fundus camera (CLARUS 500™, Carl Zeiss Meditec AG, Jena, Germany), see Fig. 1.

**Fig. 2:**
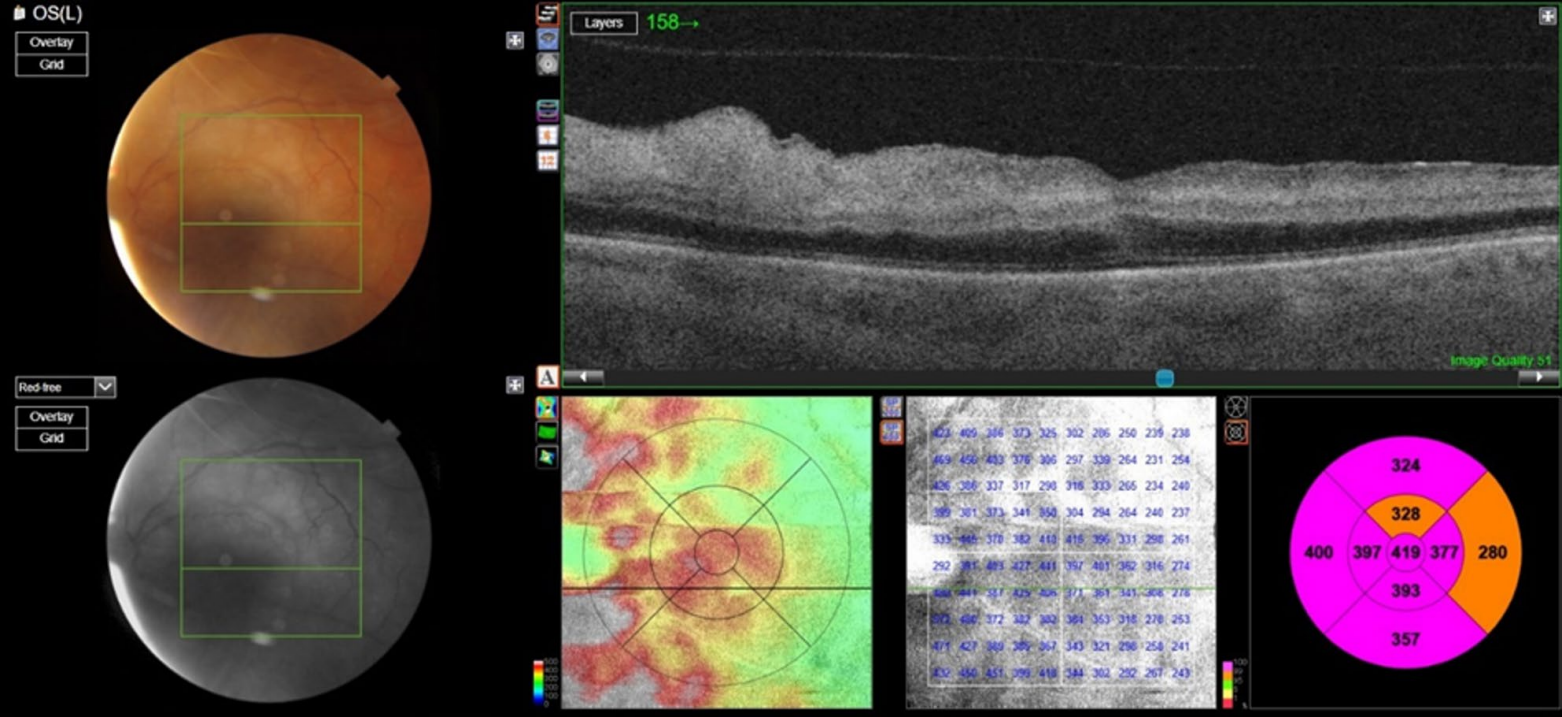
Optical coherence tomography images reveal generalized retinal thickening of the macular region, where cross-section images show hyperreflectivity in the inner retina

**Fig. 3:**
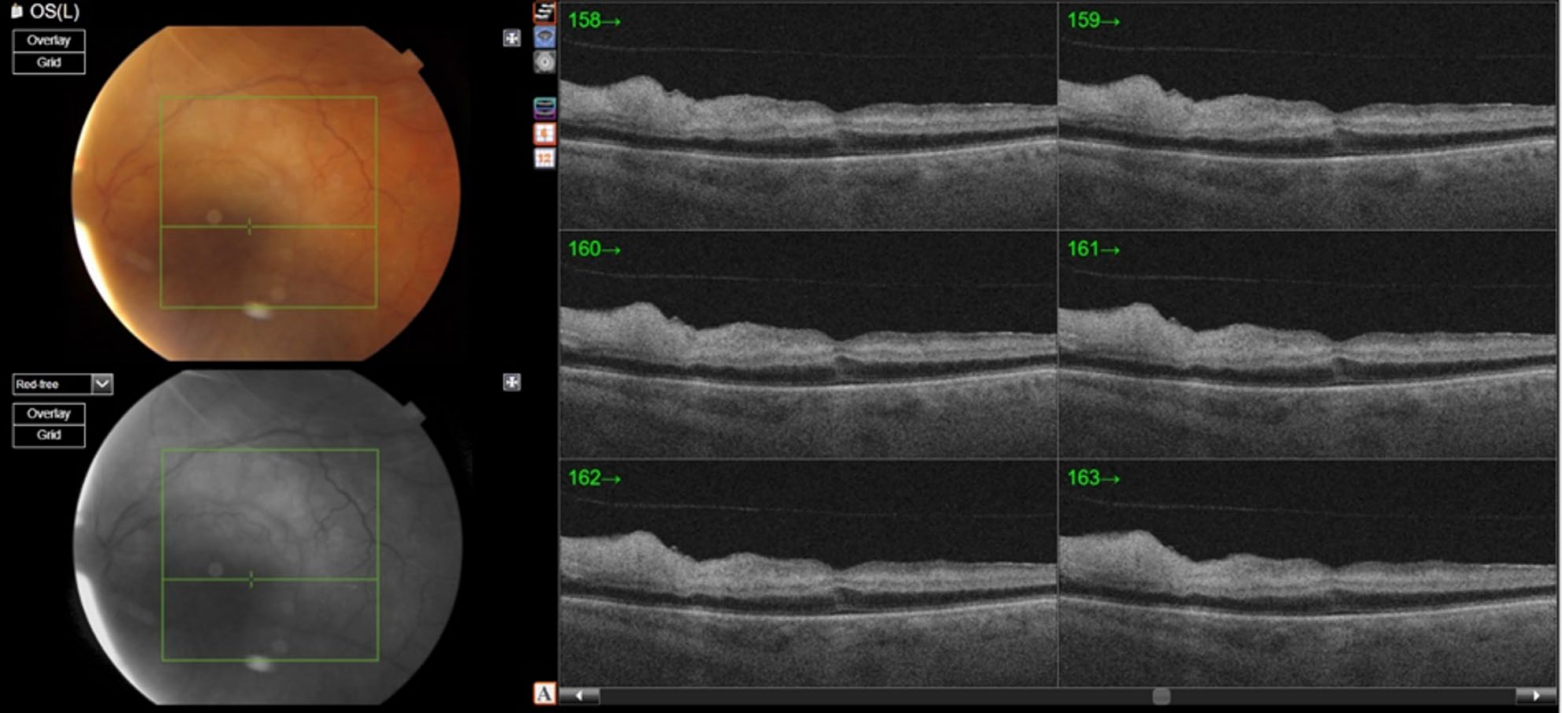
Optical coherence tomography illustrates six different cross-sectional pictures of the macula showing signs of central retinal artery occlusion

Optical coherence tomography (OCT) of the macula revealed inner retinal hyperreflectivity with increased inner retinal thickness (Figs. 2 and 3). Based on the clinical findings, a diagnosis of central retinal artery occlusion was made, and the patient was referred to the emergency department for further evaluation.

The patient underwent blood tests in the emergency department, which revealed elevated levels of erythrocyte sedimentation rate (ESR) at 53 mm/h and C-reactive protein (CRP) at 44 mg/L. However, normal values of CRP were recorded prior to TAVI, with a measurement of 3 mg/L. Hemoglobin was slightly decreased at 128 g/L, and thrombocytes were 117 10*9/L. Electrolytes remained within normal limits, but renal function showed a decline, consistent with previous assessments, with a GFR of 44 ml/min/1.7 and creatinine levels at 119 µmol/ml. Troponin levels were elevated at 200 ng/L, while coagulation parameters were normal, with a prothrombin time (INR) of 0.9 and APTT of 27 s. Vital signs indicated a blood pressure of 180/79 mmHg, a body temperature of 36.9 °C, and a heart rate of 90 bpm with a regular rhythm. Neurological examination was normal. The patient was admitted for telemetry monitoring, and a carotid artery duplex ultrasound conducted the day following admission revealed stenosis ranging from 20 to 49% (NASCET) in both the right and left internal carotid arteries, along with atherosclerotic plaque in the right external carotid artery. However, there was no stenosis observed in the common carotid artery, nor in the external carotid or common carotid artery on the left side.

## Discussion

This report details a case of central retinal artery occlusion in a 78-year-old patient with aortic stenosis following transcatheter aortic valve implantation (TAVI). While earlier studies have indicated that TAVI elevates the risk of embolic events [4], there are no prior reports of central retinal artery occlusion occurring in a patient post-TAVI.

Although the risk of major embolic events especially ischemic stroke during or after TAVI has been extensively studied [7], the effects of TAVI-related hemodynamic changes on retinal circulation and the associated risk of retinal embolic events remain poorly understood.

Cerebrovascular complications are significant contributors to both mortality and morbidity among patients receiving TAVI. Typically, these complications arise from atherosclerotic or gas microembolism, which result from the repeated manipulation of devices and catheters inside the blood vessels which can dislodge fragments from intravacscular aterosclerotic plaques which travel distal to the vessel and result in occlusion of smaller-caliber vessels [8–10]. Moreover, neurological impairment may be linked to balloon aortic valvuloplasty (BAV) and rapid ventricular pacing (RVP), which are necessary during the deployment of balloon-expandable valve types where oxygen saturation in body tissues can fluctuate. This brief and restricted duration of hypoperfusion has been suggested as a potential cause of localized ganglion cell complex (GCC) quadrant defects occurring in patients one day following TAVI. The defects observed in the GCC were demonstrated to be transient, as they vanished in all patients one month following TAVI [9]. 

In a study involving a cohort of 20 patients, researchers identified retinal abnormalities that may have arisen from unilateral retinal embolic events occurring during the early postoperative phase in 4 out of the 20 patients (20%) who underwent elective TAVI procedures. Nevertheless, no participant experienced any visual consequences after the procedure, although 1 out of 20 did suffer a clinical stroke on the sixth day following the procedure [4]. 

In another study with a total of 20 individuals, fundus photography revealed signs of embolization in three participants, retinal vessel emboli were observed in three participants, and a single cotton wool spot was observe in one patient; however, no adjacent areas of ischemia or hemorrhages were noted following TAVI. Wide-field OCTA revealed new-onset areas of focal retinal ischemia that were not visible on color fundus photography in four participants. Those findings are consistent with the impairment of the choroidal microperfusion in patients undergoing TAVI and support the hypothesis of silent embolization to the retinal vasculature during TAVI. Nevertheless, the study did not reveal any direct complications that affected the vision following TAVI in that cohort [6]. 

## Conclusion

To our knowledge, this is the first case report on visual impairment from CRAO following TAVI, although there are other reports in the literature already showing asymptomatic embolization to the retina. Our findings highlight the importance of including CRAO as one of the complications of TAVI procedure during patient counselling by the cardiologist as well as informing the neurologists of the possibility of this rare but serious complication. In situations of monocular sudden visual loss post-TAVI, an immediate referral to the eye emergency department for a full ophthalmological examination is imperative.

## Data Availability

No datasets were generated or analysed during the current study.

## Abbreviations

APTT: Activated partial thromboplastin time
AS: Aortic stenosis
BAV: Balloon aortic valvuloplasty
BCVA: Best-corrected visual acuity
CRAO: Central retinal artery occlusion
CT: Computed tomography
ESR: Erythrocyte sedimentation rate
GCC: Ganglion cell complex
GFR: Glomerular filtration rate
HM: Hand movement
INR: International normalised ratio
IU: International units
Nd: YAG Neodymium-doped yttrium–aluminum–garnet laser
NASCET: North American Symptomatic Carotid Endarterectomy Trial
NLP: No light perception
OCT: Optical coherence tomography
OCTA: Optical coherence tomography angiography
PCI: Percutaneous coronary intervention
RAPD: Relative afferent pupillary defect
RNFL: Retinal nerve fibre layer
RVP: Rapid ventricular pacing
TAVI: Transcatheter aortic valve implantation
